# Detection of norovirus infections in Denmark, 2011–2018

**DOI:** 10.1017/S0950268820000461

**Published:** 2020-02-17

**Authors:** M. R. Korcinska, K. Dalsgaard Bjerre, L. Dam Rasmussen, E. Tvenstrup Jensen, T. K. Fischer, A. Barrasa, S. Ethelberg

**Affiliations:** 1 Department of Infectious Disease Epidemiology and Prevention, Statens Serum Institut, Copenhagen, Denmark; 2 European Programme for Intervention Epidemiology Training (EPIET), European Centre for Disease Prevention and Control, Stockholm, Sweden; 3 Department of Virus and Microbiological Special Diagnostics, Statens Serum Institut, Copenhagen, Denmark; 4 Department of Research, Nordsjaellands Hospital, Hilleroed, Denmark; 5 Departments of Infectious Diseases and Global Health, University of Southern Denmark, Odense, Denmark; 6 National Centre for Epidemiology, Institute of Health Carlos III (ISCIII), Madrid, Spain; 7 Department of Public Health, Global Health Section, University of Copenhagen, Copenhagen, Denmark

**Keywords:** Epidemiology, hospital-acquired (nosocomial) infections, norovirus, Norwalk agent and related viruses, surveillance

## Abstract

Norovirus (NoV) infections occur very frequently yet are rarely diagnosed. In Denmark, NoV infections are not under surveillance. We aimed to collect and describe existing laboratory-based NoV data. National NoV laboratory data were collected for 2011–2018, including information on patient identification number, age and sex, requesting physician, analysis date and result. We defined positive patient-episodes by using a 30-day time window and performed descriptive and time series analysis. Diagnostic methods used were assessed through a survey. We identified 15 809 patient-episodes (11%) out of 142 648 tested patients with an increasing trend, 9366 in 2011 *vs.* 32 260 in 2018. This corresponded with a gradual introduction of polymerase chain reaction analysis in laboratories. The highest positivity rate was in patients aged <5 years (15%) or >85 years (17%). There was a large difference in test performance over five Danish geographical regions and a marked seasonal variation with peaks from December to February. This is the first analysis of national NoV laboratory data in Denmark. A future laboratory-based surveillance system may benefit public health measures by describing trend, burden and severity of seasons and possibly pinpoint hospital outbreaks.

## Introduction

Norovirus (NoV) is the most common cause of acute gastroenteritis and outbreaks worldwide [1]. Recent systematic reviews estimate that NoV causes 18% (95% CI 17–20%) of diarrhoeal disease worldwide [1, 2]. The exemplar clinical case is an acute and self-limiting gastroenteritis that can be managed by rehydration therapy. The virus is highly contagious and transmitted from person-to-person often indirectly via food, water, hands or contaminated surfaces. NoV can easily spread via vomit or faecal contamination, especially in the absence of appropriate hygienic conditions. The majority of the reported outbreaks in developed countries take place in healthcare facilities, including nursing homes. These outbreaks often affect vulnerable people, such as hospitalised patients or the elderly. The outbreaks are disruptive and costly to health services and are best managed through infection control measures such as improved hygiene and patient isolation [3–9]. In Northern European countries, NoV infections commonly occur in the winter months and have been referred to as ‘winter vomiting disease’ [10].

Improved diagnostics have transformed the perception of NoV from a rarely laboratory-diagnosed infection in the early 90s to the leading cause of gastroenteritis in people of all ages worldwide [11, 12]. Over the last two decades, NoV has been increasingly diagnosed by enzyme-linked immunosorbent assay (ELISA) or reverse transcription-polymerase chain reaction (RT-PCR) assay. However, the surveillance of NoV is challenging, as most infectious episodes are not diagnosed. Patients may never consult healthcare professionals and even when medical care is sought, NoV testing will rarely be conducted due to the expected self-limiting course of the disease combined with the lack of therapeutic consequences [6, 13]. There is limited information about the number of countries, where NoV is under surveillance, but for the reasons mentioned above, passive surveillance systems would likely be of limited value as early warning systems e.g. for hospital preparedness planning during excessive NoV seasons. However, a study of laboratory surveillance data from England and Wales has suggested a potential value of combining different information sources (information about the infection aetiology, seasonality, virology factors etc.) into a virus early warning system [14].

In Denmark, a country with 5.8 million inhabitants, NoV is thought to be a common infection. A recent burden of disease study estimated 185 000 cases (incidence 3203 per 100 000 inhabitants), 26 deaths and 485 disability-adjusted life years to have occurred in 2017 [15]. Moreover, NoV is a frequent cause of outbreaks in healthcare institutions, the most common aetiology among foodborne infections [16–20] and most frequent cause of foodborne outbreaks, responsible for 33% of known such outbreaks in 2018 [21]. NoV is currently not covered by national disease surveillance and there are no national guidelines or recommendations covering the practices at regional laboratories, thus there is no information on the laboratory practices. We took advantage of the recently implemented Danish Microbiology Database (MiBa) [22] to compile national diagnostic data from 2011 to 2018, in order to learn more about the epidemiology of the infections in Denmark. In addition, we conducted a survey to investigate the current laboratory testing practices.

## Methods

### Data source

In Denmark, hospital services, including microbiological analyses, are conducted within five geographic administrative regions. Diagnosis of NoV in Denmark is undertaken at publicly financed clinical microbiology laboratories (KMAs). Currently, there are 10 KMAs, which use different diagnostic methods and laboratory information systems. During the study period 12 KMAs existed (including the reference laboratory) as some laboratories have recently been merged. In 2010, the MiBa was created, which contains copies of all electronic KMA analysis reports in Denmark, which can later be identified by three parameters: an MiBa identification number, the patient's unique civil registry number (CPR) and the report ID number used by the KMA. All Danish residents are registered by a unique population register (CPR) number [23], which allows for the elimination of duplicate recordings and for linking of data between registries. Although all KMAs provide information in coded form, a national standard for coding is not applied for all variables. Some elements in the reports follow national coding standards while other elements follow local standards. To extract data on NoV diagnostics from the MiBa, we searched for all relevant codes and remapped them through a standardisation process. NoV laboratory data were compiled for the period of 2011–2018 including information on the requesting physician, requested analysis, results and person information. Information about Danish general and age-specific sizes of the population was obtained from Denmark Statistics [24]. As the laboratory diagnostic methods were changing over time, the study period was divided into two intervals of 4 years (2011–2014 and 2015–2018) for better data presentation.

### Test-episode and patient-episode definitions

Laboratory analysis was performed using an antigen ELISA or by PCR. Some laboratories use the GeneXpert Rapid Point of Care Test, which is based on PCR and is performed in the laboratory. Individuals were uniquely identified by their CPR number. We excluded tests with inconclusive results as well as individuals with invalid or temporary CPR numbers, which is frequently given to tourists. Many individuals had several tests performed within a short time. To address multiple episodes of testing in the same patient, we considered tests performed within a 30-day time period as relating to the same episode of illness. Thus, we defined a test-episode as the series of one or more tests done on the same individual within a 30-day period starting on the date of the first sample. Test-episodes were either positive or negative based on the results of tests: one or more positive tests in a test-episode defined a patient-episode, while a series of only negative tests defined a negative test-episode. To assess the sensitivity of the 30-day time window, we also explored 60-days and 1-year time windows.

### Data analysis

Descriptive analyses in terms of time, place and person were performed. The number of test-episodes, patient-episodes and incidences hereof were calculated by sex, age group and region. We counted the proportion of positive and negative test-episodes in addition to the test positivity rate. We assessed the significance of the increasing trends with the Cuzick test. We described and determined the duration of each season between 2011/2012 and 2017/2018. We used time series analysis and calendar plots to explore disease count distribution by week. Data between 2011 and 2018 were modelled by using Poisson regression, adjusted for seasonality using sine and cosine functions. The observed seasons were roughly classified as large (L) and small (S) according to their size related to the Poisson model. All calculations were performed by using STATA version 14 (StataCorp, Texas, USA).

### Laboratory practice survey

To describe testing practices, a questionnaire was sent in May 2018 by email to representatives from all KMAs. The questionnaire included open and closed questions about the methodology and recommendations used in each laboratory during the past 7 years (2011–2017). We asked KMA representatives about how the test for NoV was performed (individually or as a part of packages for multiple pathogens) and about the methodology used in the laboratories/hospitals (RT-PCR, ELISA or the GeneXpert). Participants were also asked about any changes in the methods used during the study period. Finally, we asked if laboratories followed recommendations regarding the number of samples analysed per individual, the number of patients tested per outbreak group or regarding test of individuals from risk groups (age group, sex, comorbidity or immunosuppression).

## Results

### Descriptive analysis: age, sex, place, and test performance

Between 2011 and 2018, a total of 177 397 laboratory analyses for NoV could be found and extracted from MiBa, of which 19 955 (11%) were positive. They constituted 142 648 test-episodes (series of tests on the same individual) of which 15 809 (11%) were positive, representing patient-episodes. The number of tests within the test-episodes varied, 83% of patients had one sample analysed, 12% had two, 5% had three or more (max. 15 samples). In total, 24 449 test-episodes consisted of two tests performed on the same individual. In 4% of these, the test results were diverging. In total, 7658 test-episodes consisted of three or more tests. In total, 1% had diverging results. Defining episodes by 60-days and 365-days' time windows, these results changed only marginally. With the 60-days window there were 139 071 test-episodes of which 15 688 were positive (11%) and with a 365-days window there were 129 828 test-episodes of which 15 450 were positive (12%). The following results are therefore based on a 30-days window. Table 1 shows the distribution of the number of tests, test-episodes, tests result and mean population size per region during two different periods. The total number of tests doubled from 62 705 during the first period to 114 692 in the second period whereas the positivity rate decreased from 14.5% to 9.5%. Most tests were done in the Capital Region (65% of all tests) and the least in the Central Region (2%). During 2015–2018, the Capital Region performed 859 tests per 100 000 population whereas the Central Region performed 47 tests per 100 000 population.

**Table 1.** Number of NoV tests, positive tests, test-episodes, patient-episodes, population and incidence per person-year in two time periods using a 30 days window between test series

Region	Population (average)	Tests	Positive tests	Test positive rate	Test-episodes	Patient-episodes	Test-episodes positive rate	Test-episodes incidence^a^	Patient-episodes incidence^a^
2011–2014									
Northern	580 436	3966	596	15.0	3103	504	16.2	134	22
Central	1 269 757	66	23	34.8	56	19	33.9	1	0
Southern	1 201 558	15 601	2407	15.4	12 798	1932	15.1	266	40
Zealand	817 443	2403	410	17.1	2217	361	16.3	68	11
Capital	1 726 199	40 669	5652	13.9	27 138	3875	14.3	393	56
Total	5 595 394	62 705	9088	14.5	45 312	6691	14.8	202	30
2015–2018									
Northern	587 828	3867	641	16.6	3625	546	15.1	154	23
Central	1 307 024	2763	627	22.7	2477	551	22.2	47	11
Southern	1 217 744	19 009	2212	11.6	15 412	1712	11.1	316	35
Zealand	832 279	14 626	1181	8.1	13 511	1104	8.2	406	33
Capital	1 812 458	74 427	6206	8.3	62 311	5205	8.4	859	72
Total	5 757 333	114 692	10 867	9.5	97 336	9118	9.4	423	40
2011–2018									
Northern	584 397	7833	1237	15.8	6728	1050	15.6	144	22
Central	1 290 472	2829	650	23.0	2533	570	22.5	25	6
Southern	1 210 109	34 610	4619	13.3	28 210	3644	12.9	291	38
Zealand	824 994	17 029	1591	9.3	15 728	1465	9.3	238	22
Capital	1 775 960	115 096	11 858	10.3	89 449	9080	10.2	630	64
Total	5 685 931	177 397	19 955	11.2	142 648	15 809	11.1	314	35

a Incidence per 100 000 person-year.

**Fig. 1.**
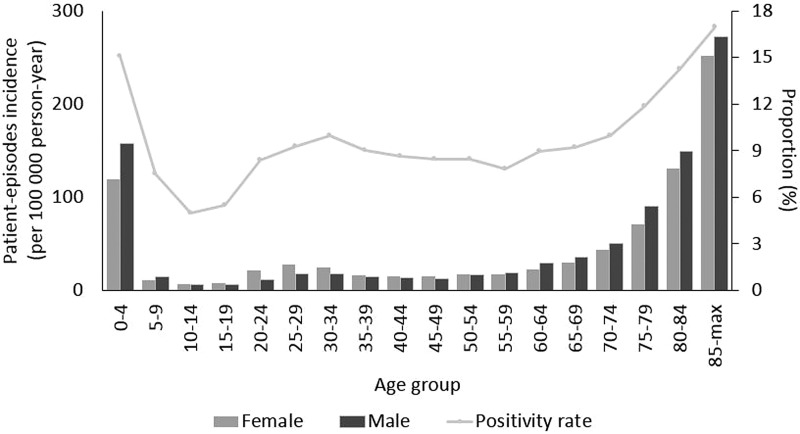
NoV patient-episode incidence by sex and age group and overall proportion of patient-episodes and test-episodes (positivity rate) by age group, Denmark 2011–2018.

**Fig. 2.**
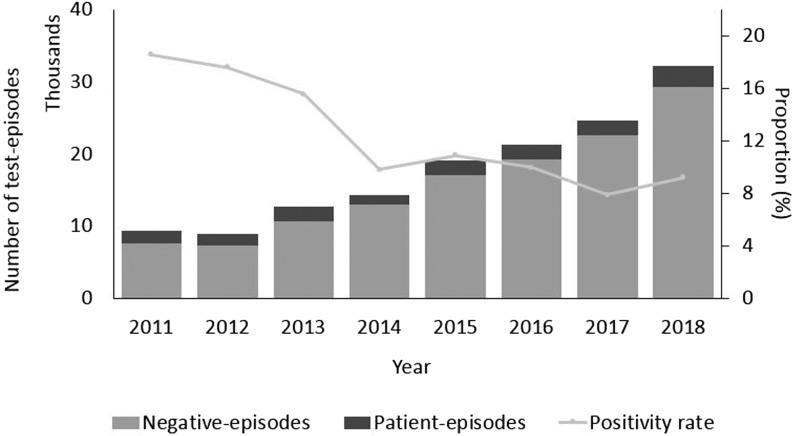
Number of test episodes, patient-episodes and negative test-episodes per year and proportion of patient-episodes and test-episodes (positivity rate), Denmark 2011–2018.

The highest proportion of positive test episodes were in patients less than 5 years old (15%) or older than 85 years (17%) (Fig. 1). There was a noticeable increase in incidence among older age groups (60 and more years old, *P* < 0.05). Incidence was larger among females than males in the age group between 20 and 34 years. In contrast, among the elderly above 60 years of age incidence was larger among males than females. The largest difference in sex incidence was in males (157 per 100 000 person-years) compared to females (119) in the youngest age group (Fig. 1). During the study period, due to an increasing trend in the number of test-episodes performed (from 9366 in 2011 to 32 260 in 2018, *P* < 0.05) (Fig. 2), the positivity rate decreased as the number of positive test-episodes only increased slightly by year.

### Laboratory practise survey

Nine out of 10 present laboratories answered the survey. Existing NoV data showed that 89% of laboratory results were obtained using RT-PCR to detect virus RNA. During the study period, some laboratories introduced in-house RT-PCRs. Methods used in the individual laboratories as well as introduced changes including approximate dates are summarised in Table 2. The introduction of RT-PCR in some laboratories in Zealand and Central Regions resulted in a slight increase in the number of tests performed. The regional laboratory in the Northern Region introduced RT-PCR in 2013 that surprisingly did not result in an increase as was observed in the other laboratories. However, in 2015, after the introduction of the GeneXpert test method, the number of tests almost doubled. There was a concurrent decrease in test numbers performed at the reference laboratory, which is placed at SSI, in the Capital Region because this laboratory has mainly been diagnosing NoV in cases related to suspicions of foodborne outbreaks from 2015 onwards. In January 2016, one of the Capital Region laboratories, placed in the tertiary hospital ‘Rigshospitalet’ included NoV analyses in a ‘package’ to diagnose gastroenteritis, also including tests for other viruses and bacteria. As a result, the number of reported tests doubled in the following years.

**Table 2.** Number of NoV tests in different regions with comment about main changes in testing methods in regional laboratories, Denmark 2011–2018

Region	No. of KMAs active during study period	Year								Comment
		2011	2012	2013	2014	2015	2016	2017	2018	
Northern	1	1102	1208	985	671	1173	719	822	1153	**January 2010** – Norovirus antigen test (EIA Test from Oxoid); **July 2013** – Introduction of RT-PCR (Altona Diagnostics); **January 2015** – Change to GeneXpert (Xpert Norovirus, Cepheid)
Central	2	0	0	0	66	522	707	580	954	**October 2014** – Introduction of in-house RT-PCR; **March 2015** – Expanding testing method for whole region
Southern	3	3351	4068	4154	4028	4680	4012	4463	5854	**Before 2013** – Use of individual test and package for viral diseases (NoV + rotavirus); **After 2013** – Introduction of PCR package for viral diseases (NoV + rotavirus + adenovirus + sapovirus + astrovirus); **January 2018** – Introduction of GeneXpert
Zealand	1	0	115	749	1539	2456	2977	3716	5477	**September 2012** – Introduction of in-house RT-PCR
Capital	4	4312	3855	9130	10 264	13 556	15 417	19 150	24 241	**2013** – Introduction of in-house RT-PCR in one laboratory; **2010-2016** – Use of package for viral diseases (NoV + rotavirus + adenovirus) by RT-PCR (BD-max); **January 2016** – Change to gastroenteritis package (virus + bacteria) by RT-PCR (C-gene)
Reference laboratory	1	6256	4494	1384	974	517	497	401	648	**January 2015** – Diagnostic performed mainly due to foodborne outbreaks
Total	12	15 021	13 740	16 402	17 542	22 904	24 329	29 132	38 327	

Recommendations of the laboratories in connection with nosocomial outbreaks are not uniform. For example, some of the hospitals (in Zealand and Capital Regions) sample only the first patient during outbreaks, whereas other hospitals (Southern and Capital Regions) reported to be testing all patients affected by an outbreak. In addition, varying instructions were identified regarding the recommended number of samples to be tested: most laboratories recommended taking 1–2 samples per patient whereas a few hospitals in the Capital Region recommended three samples per patient.

### Seasonal variation

**Fig. 3.**
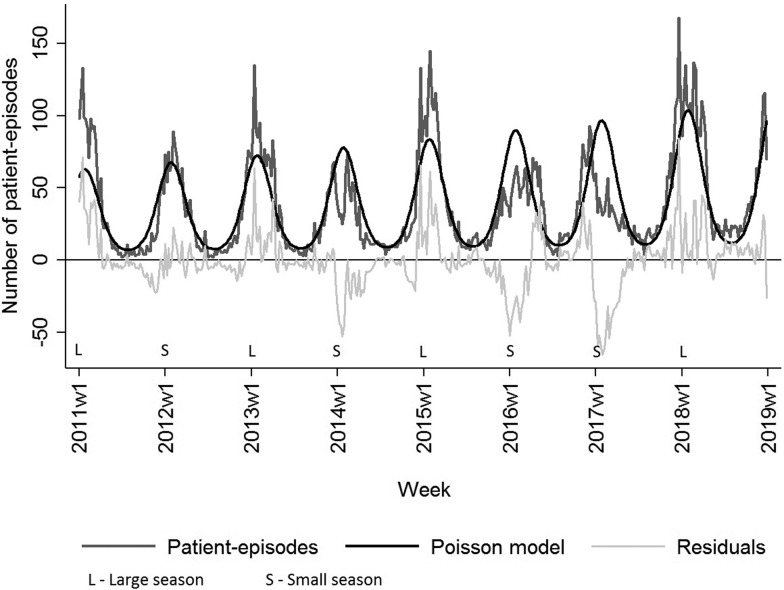
Number of weekly NoV laboratory-diagnosed patient-episodes, number of modelled patient-episodes and residuals hereof, Denmark 2011–2018. NoV seasons are classified as larger than modelled or smaller than modelled.

Figure 3 shows the weekly distribution of the NoV patient-episodes. There was a very clear seasonal variation in NoV infection with the majority of patient-episodes occurring in the winter months (December–February), peaking around weeks 4–5. The Poisson regression model fitted to the seasonal data highlights how the patient-episodes increase at a similar time each year, around week 45 (November) and decrease in week 14 (March). The seasonal pattern of the NoV patient-episodes seems to change biannually, while the number of patient-episodes seems to rotate between ‘large’ and ‘small’ seasons. Correspondingly, seasons from 2011 to 2018 can be classified as 2011/2012 (small), 2012/2013 (large), 2013/2014 (small), 2014/2015 (large), 2015/2016 (small), 2016/2017 (small) and 2017/2018 (large). Seasons 2015/2016 and 2016/2017 were atypical in their shape: longer by 2–3 weeks and less intensive in comparison with other seasons. The last season, 2017/2018 was more intensive with the highest number of patient-episodes (Fig. 3).

## Discussion

For the first time in Denmark, laboratory-based data on diagnosed NoV tests were compiled and described in terms of time, place and person. Over the study period, the number of NoV tests increased annually. Test frequency varied highly between different hospitals in Denmark. The majority of tests were performed in children and elderly patients. These groups also had the highest proportions of positive tests, suggesting that the real incidence of disease is higher in these age groups or alternatively it might reflect the hospitalisation age group incidence. There was a marked seasonal variation, with the season running from the end of December to February.

The epidemiological patterns are similar to what has been reported from other countries. Even though NoV is associated with acute gastroenteritis among children, recent studies show that other age groups such as the elderly are also at high risk if living in institutional settings. NoV outbreaks are common in hospitals or nursing homes [9, 25, 26]. In Denmark, data on healthcare settings outbreaks are not collected. However, if national laboratory data could be linked to the national hospitalisation database, this could in theory allow us to monitor the nosocomial outbreaks. Such information could be of value to hospitals, working on reducing the impact of NoV outbreaks.

The increasing test activity in Denmark could be explained by the gradual introduction of RT-PCR and the fact that more tests are performed as a part of multi-pathogen testing for gastroenteritis. Laboratory identification of NoV is useful for guidance of patient isolation measures as well as the overall reduction of NoV transmission through infection control and public health interventions [27, 28]. Currently in Denmark, nine of 10 KMAs are using an RT-PCR to diagnose NoV. In the near future the rapid point-of-care test popularity may increase, which also can play a role in increasing the number of tests. However, if such testing is introduced as bedside testing, these cases might escape laboratory-based surveillance.

We saw a strong seasonal distribution. In Denmark, foodborne NoV outbreaks are reported throughout the year, however, the known foodborne outbreak cases constituted only a small fraction of the cases in our dataset. A marked seasonal distribution with a peak in the colder months of cases, and to a lesser extent of outbreaks, was described in a recent analysis of published papers [29]. It also showed that northern European countries generally report that most cases occur in December–February. The seasonal patterns may also be affected if the viruses in circulation are changing [1, 2, 12]. The NoV genome has a high mutation rate and NoV may undergo genetic shifts which result in new strains that are able to infect partially immune hosts that already experienced the old strains. A review of NoV strains circulating over the last decades showed emergent strains replacing previous ones, resulting in new global epidemics, appearing every 2–4 years [26]. The time and scale of NoV seasonal activity in Denmark differs from season to season. One explanation of this pattern could be that immunity in the population, after a large season, has increased resulting in fewer cases during the next season. Changes in the dominating genotype render the population renewed susceptible. In late 2014, researchers from Asia reported a major shift from the predominant GII.4 to the novel GII.P17-GII.17 NoV strain (GII.17 Kawasaki 2014). The novel GII.P17-GII.17 NoV variant circulated widely in Europe in the season 2015–2016 [30, 31]. In Denmark, the new GII.17 Kawasaki 2014 strain was identified for the first time in late 2015 and first identified in connection with a foodborne outbreak early in 2016 [32], which supports the theory of the biennial changes. In many developed countries, as well as in Denmark, outbreaks in healthcare settings are most often caused by NoV genogroup II genotype 4 (GII.4). This genotype is mostly associated with the elderly population [33, 34]. The absence of routine surveillance on the gastroenteritis outbreaks in hospitals limits the information about changes in virus variants circulating in healthcare settings.

Although this study covers data available in the national MiBa database from 2011 through 2018, we acknowledge the possibility that not all diagnosed NoV cases were identified. Timely monitoring of the cases could help identify the beginning of the season, which would likely improve the prevention and control measures by public health and healthcare settings personnel, for instance help with planning for increased healthcare usage in facilities or alert messages to the public. It would be important to further investigate the NoV seasonal activity after passive surveillance system implementation. A passive surveillance system for NoV in Denmark is anticipated to be approved within the near future. This automated data collection should provide timely and relevant information for public health action; in the future, if combined with for instance the hospital discharge database, it might assist in identifying and managing further outbreaks.

In conclusion, this study suggests that the introduction of an automated passive NoV laboratory-based surveillance system in Denmark would be helpful both for long-term trend analyses and for managing outbreaks within hospitals and healthcare facilities. If information on hospitalisation status could be included in the surveillance, it would be a valuable tool in the managing of hospital outbreaks and the evaluation of their impact. Likewise, timely identification of the beginning of and the strength of an annual NoV season could provide a preparedness signal to alert healthcare facilities. This could guide implementation of control measures and thus aid in reducing the substantial morbidity as well as the economic impact of NoV outbreaks.
